# Astaxanthin prevents and reverses diet-induced insulin resistance and steatohepatitis in mice: A comparison with vitamin E

**DOI:** 10.1038/srep17192

**Published:** 2015-11-25

**Authors:** Yinhua Ni, Mayumi Nagashimada, Fen Zhuge, Lili Zhan, Naoto Nagata, Akemi Tsutsui, Yasuni Nakanuma, Shuichi Kaneko, Tsuguhito Ota

**Affiliations:** 1 Department of Cell Metabolism and Nutrition, Brain/Liver Interface Medicine Research Center, Kanazawa University, Kanazawa, Ishikawa 920-8640, Japan; 2 Department of Disease Control and Homeostasis, Kanazawa University Graduate School of Medical Science, Kanazawa, Ishikawa 920-8640, Japan; 3 Department of Pathology, Kanazawa University Graduate School of Medical Science, Kanazawa, Ishikawa 920-8640, Japan

## Abstract

Hepatic insulin resistance and nonalcoholic steatohepatitis (NASH) could be caused by excessive hepatic lipid accumulation and peroxidation. Vitamin E has become a standard treatment for NASH. However, astaxanthin, an antioxidant carotenoid, inhibits lipid peroxidation more potently than vitamin E. Here, we compared the effects of astaxanthin and vitamin E in NASH. We first demonstrated that astaxanthin ameliorated hepatic steatosis in both genetically (*ob/ob*) and high-fat-diet-induced obese mice. In a lipotoxic model of NASH: mice fed a high-cholesterol and high-fat diet, astaxanthin alleviated excessive hepatic lipid accumulation and peroxidation, increased the proportion of M1-type macrophages/Kupffer cells, and activated stellate cells to improve hepatic inflammation and fibrosis. Moreover, astaxanthin caused an M2-dominant shift in macrophages/Kupffer cells and a subsequent reduction in CD4^+^ and CD8^+^ T cell recruitment in the liver, which contributed to improved insulin resistance and hepatic inflammation. Importantly, astaxanthin reversed insulin resistance, as well as hepatic inflammation and fibrosis, in pre-existing NASH. Overall, astaxanthin was more effective at both preventing and treating NASH compared with vitamin E in mice. Furthermore, astaxanthin improved hepatic steatosis and tended to ameliorate the progression of NASH in biopsy-proven human subjects. These results suggest that astaxanthin might be a novel and promising treatment for NASH.

Non-alcoholic fatty liver disease (NAFLD) and insulin resistance coexist frequently in subjects with obesity and type 2 diabetes^1^,^2^, and affect 20–30% of the general population and 70–80% of obese and diabetic subjects^3^. It has become increasingly evident that hepatic inflammation and/or fibrosis could be caused by excessive hepatic lipid accumulation followed by lipid peroxidation. This ectopic fat or lipotoxicity in the liver induces an innate immune response with the subsequent recruitment of immune cells such as macrophages and T cells, which leads to the development of insulin resistance and NASH^4^. Previously, we developed a cholesterol- and saturated fatty acid-induced model of lipotoxic NASH that replicated the pathophysiological features of human NASH successfully, and found that excessive hepatic lipid accumulation promoted the activation of macrophages/Kupffer cells to exacerbate insulin resistance and hepatic inflammation and fibrogenesis^5^.

Although both insulin resistance and oxidative stress are pivotal for the progression of NASH, no standard therapy for NASH has been established^4^,^6^. Many agents have been tested for the management of NASH, with disappointing results, the antioxidant vitamin E is somewhat efficacious. In the largest randomized controlled trial performed to date, the PIVENS trial demonstrated that vitamin E reduced hepatic steatosis and lobular inflammation in adult patients with NASH^7^. However, there is a continuing need for additional and more effective therapies for patients with NASH.

Micronutrient antioxidants, such as vitamins and carotenoids, are depleted severely in the serum and liver tissue of patients with chronic liver diseases and cirrhosis^8^,^9^. Meanwhile, elevated alanine aminotransferase (ALT) levels were associated with decreased antioxidant, particularly carotenoids such as α- and β-carotene, and lutein levels in a large population-based study^10^. More recently, it was reported that plasma vitamin E and carotenoid levels were decreased in patients with NASH^11^. Importantly, carotenoids inhibit lipid peroxidation as potently as does vitamin E^12^,^13^, but carotenoid supplementation has not been used widely as an antioxidant therapy for the treatment of NASH. Recently, we have shown that β-cryptoxanthin, an antioxidant carotenoid, inhibits the progression of NASH in mice^14^,^15^. However, the effects of carotenoid compared with vitamin E are still not clear.

Astaxanthin is a xanthophyll carotenoid found in marine organisms, including salmon, shrimp, crustaceans, and algae such as *Haematococcus pluvialis*^16^. Astaxanthin inhibits lipid peroxidation 100–500-fold more strongly than vitamin E *in vitro*^17^, and has several-fold greater free radical antioxidant activity than do vitamin E and β-carotene^18^. The administration of astaxanthin inhibits carbon tetrachloride-induced lipid peroxidation in the rat liver^19^, and suppresses the upregulation of fibrogenic genes in hepatic stellate cells (HSCs) by blocking TGF-β/Smad3 signaling^20^. In addition, astaxanthin prevents diet-induced obesity and hepatic steatosis in mice^21^ and ameliorates insulin resistance by protecting myocytes from oxidative stress^22^.

Therefore, we hypothesized that the administration of astaxanthin would inhibit the progression of NASH by suppressing lipotoxicity-induced oxidative stress and the subsequent lipid peroxidation and insulin resistance. Here, the preventative and therapeutic effects of astaxanthin and vitamin E in a lipotoxic model of NASH were compared. The data revealed that astaxanthin prevented and reversed hepatic insulin resistance and NASH similarly or more potently than did vitamin E by regulating macrophage and T-cell accumulation as well as the M1/M2 status of macrophages/Kupffer cells in the liver of mice. Furthermore, astaxanthin alleviated the progression of NASH in humans.

## Results

### Astaxanthin Alleviated Hepatic Steatosis in Obese Mice and Decreased Lipid Accumulation in Hepatocytes

**Figure 1:**
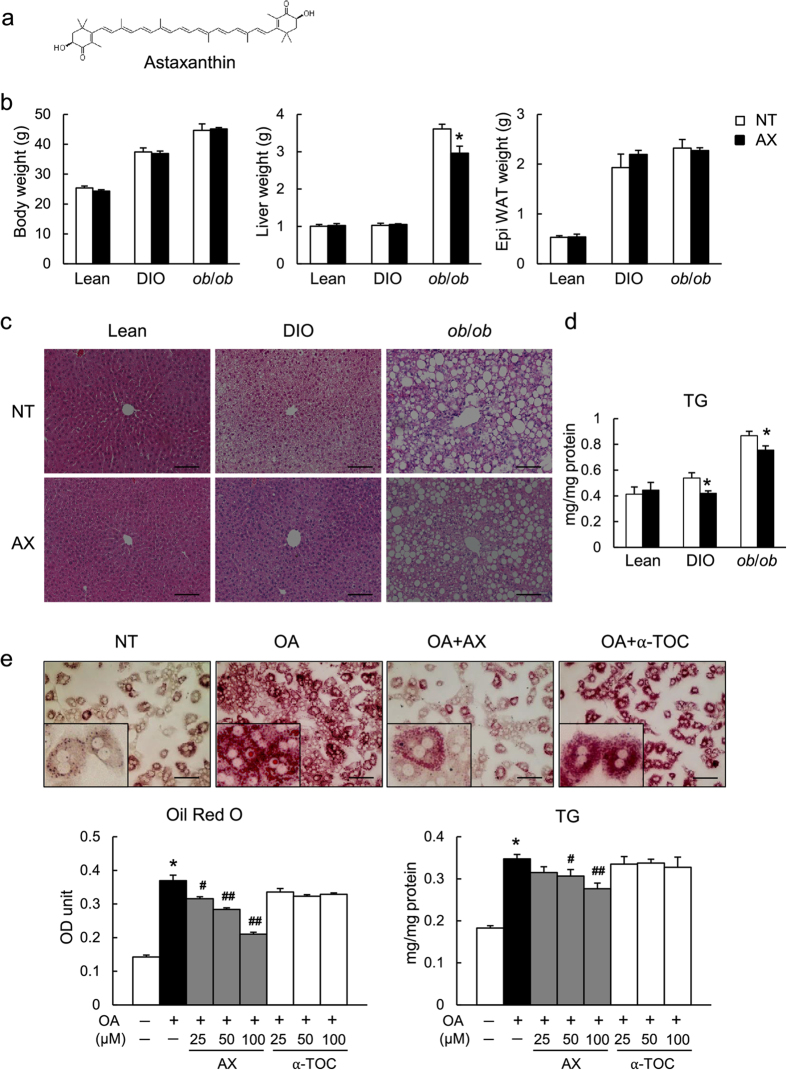
Astaxanthin reduced hepatic steatosis in DIO and *ob/ob* mice and decreased lipid accumulation *in vitro*. (**a**) The chemical structure of astaxanthin. (**b**) The body weights and tissue weights of mice (*n* = 5–8). NT, no treatment; AX, astaxanthin treatment. (**c)** Representative hematoxylin and eosin (H&E)-stained liver sections. Scale bars = 100 μm. (**d**) Hepatic TG content (*n* = 5–8). *P < 0.05 vs. control group. (**e**) Oil Red O staining of cultured primary hepatocytes and cellular TG levels (*n* = 6). *P < 0.01, vs. control incubation; ^#^*P* < 0.05, ^##^*P* < 0.01 vs. oleic acid (OA)-treated cells.

To assess the effect of astaxanthin on hepatic steatosis, high-fat diet (HFD)-induced obese (DIO) and genetically obese (*ob/ob*) mice were treated with astaxanthin. Figure 1a shows the chemical structure of astaxanthin. After 10 weeks of feeding, astaxanthin administration reduced hepatic steatosis and triglyceride (TG) accumulation significantly in both DIO and *ob/ob* mice, even though weight and adiposity were not affected by astaxanthin (Fig. 1b,d). To further clarify the effect of astaxanthin on lipid accumulation *in vitro*, primary hepatocytes were incubated with either astaxanthin or α-tocopherol, a lipophilic antioxidant that is efficacious when used to treat NAFLD, in the presence of oleic acid. Incubation with astaxanthin, but not α-tocopherol, resulted in dose-dependent decrease in TG accumulation, as assessed by Oil Red O staining and the cellular TG content in lipid-loaded primary hepatocytes (Fig. 1e). Next, quantitative real-time PCR (qPCR) analysis was performed to elucidate the mechanism by which astaxanthin decreased lipid accumulation in hepatocytes (Fig. S1a). Astaxanthin treatment did not affect the mRNA levels of lipogenic and fatty acid oxidation genes, while expression of *Cd36*, a key regulator of lipid uptake, was increased significantly by oleic acid treatment, and decreased by astaxanthin in a dose-dependent manner (Fig. S1a). Moreover, the phosphorylation levels of p38 MAPK and c-Jun in oleic-acid-loaded hepatocytes were unaffected by astaxanthin (Fig. S1b). To assess whether the attenuation of lipid accumulation by astaxanthin is associated with protection against lipotoxicity, hepatocytes were co-incubated with palmitic acid and astaxanthin. However, astaxanthin had little effect on apoptosis, as assessed by cleavage of caspase-3, and viability in palmitic acid-loaded hepatocytes (Fig. S2). Taken together, these results suggest that astaxanthin reduced lipid accumulation by decreasing lipid uptake, and therefore improved simple fatty liver.

### Astaxanthin Improved Dyslipidemia and Liver Dysfunction in a NASH Model

To determine the most effective doses of astaxanthin on diet-induced NASH, C57BL/6J mice were fed different diets for 12 weeks (normal chow [NC], NC containing 0.0067% or 0.02% astaxanthin, high-fat, cholesterol, and cholate diet [CL], or CL containing 0.0067% or 0.02% astaxanthin). Treatment with astaxanthin ameliorated liver pathology and decreased plasma aspartate aminotransferase (AST) and ALT levels in diet-induced NASH in a dose-dependent manner (Fig. S3a and S3b). Astaxanthin accumulated in various tissues after consumption of the CL diet containing 0.02% (w/w) of this material (Fig. S3c); the astaxanthin concentrations were higher in the spleen, heart, and liver than in other tissues, suggesting that astaxanthin accumulates in the liver and ameliorates NASH. The effects were more prominent in the 0.02% astaxanthin-treated group; therefore, subsequent experiments were performed using that dose (named the CL+AX group).

Next, we compared the effects of astaxanthin and vitamin E as similar lipophilic antioxidants on the prevention of NASH. The metabolic parameters of mice after 12 weeks on the CL diet are shown in Table 1. Astaxanthin decreased plasma TG, total cholesterol (TC), non-esterified fatty acid (NEFA), AST, and ALT levels significantly in CL mice. In contrast, vitamin E tended to decrease TG and TC levels, but did not affect NEFA, ALT, and AST levels. Bodyweight, food intake, and liver weight were unaffected by astaxanthin or vitamin E in both NC and CL mice. These results suggest that astaxanthin improved dyslipidemia and liver dysfunction in NASH mice.

### Astaxanthin Prevented the Development of Hepatic Steatosis by Suppressing Lipogenic Gene Expression

**Figure 2:**
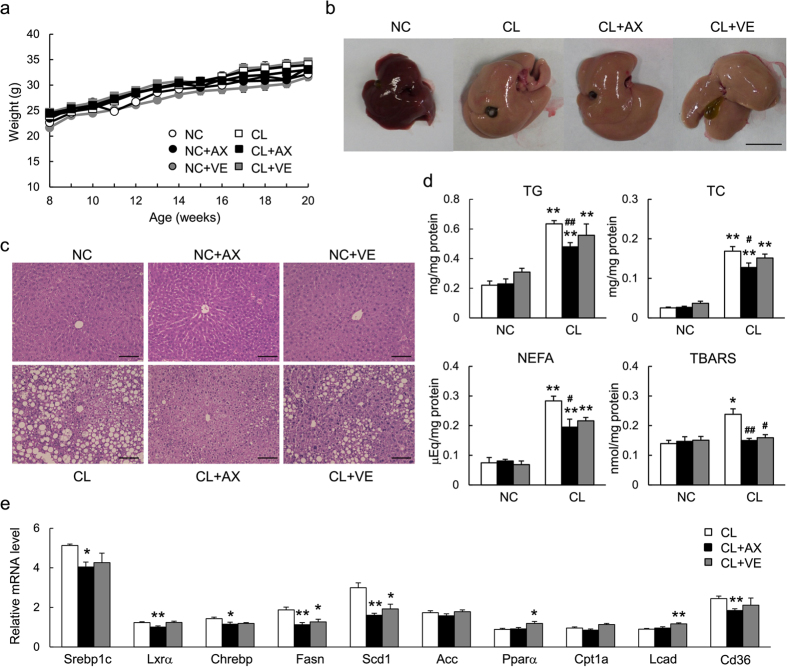
Astaxanthin prevented the development of hepatic steatosis in NASH mice. (**a**) Weight gain in mice. (**b**) Representative photographs of liver. Scale bars = 1 cm. (**c**) Representative H&E-stained liver sections. Scale bars = 100 μm. (**d**) Hepatic TG, TC, NEFA, and TBARS contents (*n* = 5–8). *P < 0.05, **P < 0.01 vs. the NC diet; ^#^*P* < 0.05, ^##^*P* < 0.01 vs. the CL-diet-fed group. (**e**) mRNA expression of lipogenic and fatty acid oxidation genes in the livers of mice (*n* = 8). *P < 0.05, **P < 0.01 vs. the CL group.

Mice in each group had similar bodyweights (Fig. 2a) and consumed similar quantities of food (Table 1). However, liver size was increased significantly by CL diet feeding, and was unaffected by astaxanthin and vitamin E administration (Fig. 2b). Histological analysis revealed severe lipid accumulation in the livers of CL mice, which was decreased markedly by astaxanthin and decreased slightly by vitamin E at 20 weeks of age (Fig. 2c). Consistent with these histological findings, CL mice exhibited significantly increased hepatic TG, TC, and NEFA levels compared with NC-fed mice, whereas astaxanthin administration reduced lipid accumulation significantly in the CL group (Fig. 2d). However, vitamin E treatment did not reduce hepatic lipid levels. The levels of thiobarbituric acid reactive substances (TBARS), an index of lipid peroxidation and oxidative stress, in the liver were increased by the CL diet feeding, revealing exaggerated lipid peroxidation in the livers of NASH mice. Both astaxanthin and vitamin E treatment decreased hepatic lipid peroxidation (Fig. 2d).

During the development of steatohepatitis, the expression of lipogenic regulator genes, including *Srebp1c*, *Lxra*, *Chrebp*, and fatty acid synthesis genes, including *Fasn and Scd1*, was increased significantly in the livers of CL compared with NC mice (Fig. 2e, Fig. S4a). Treatment with astaxanthin suppressed the expression of these lipogenic genes, whereas vitamin E did not alter or slightly decreased gene expression (Fig. 2e). By contrast, astaxanthin had little effect on the expression of genes related to fatty acid oxidation, whereas vitamin E administration increased the expression of *Ppara* and *Lcad* in CL mice (Fig. 2e, Fig. S4a). On the other hand, the upregulated expression of *Cd36* by CL diet was downregulated by astaxanthin, and unaffected by vitamin E (Fig. 2e, Fig. S4a). These results suggest that astaxanthin suppressed lipogenesis and lipid uptake to reduce lipid accumulation in the liver of NAFLD/NASH mice.

### Astaxanthin Improved Glucose Intolerance and Insulin Resistance

**Figure 3:**
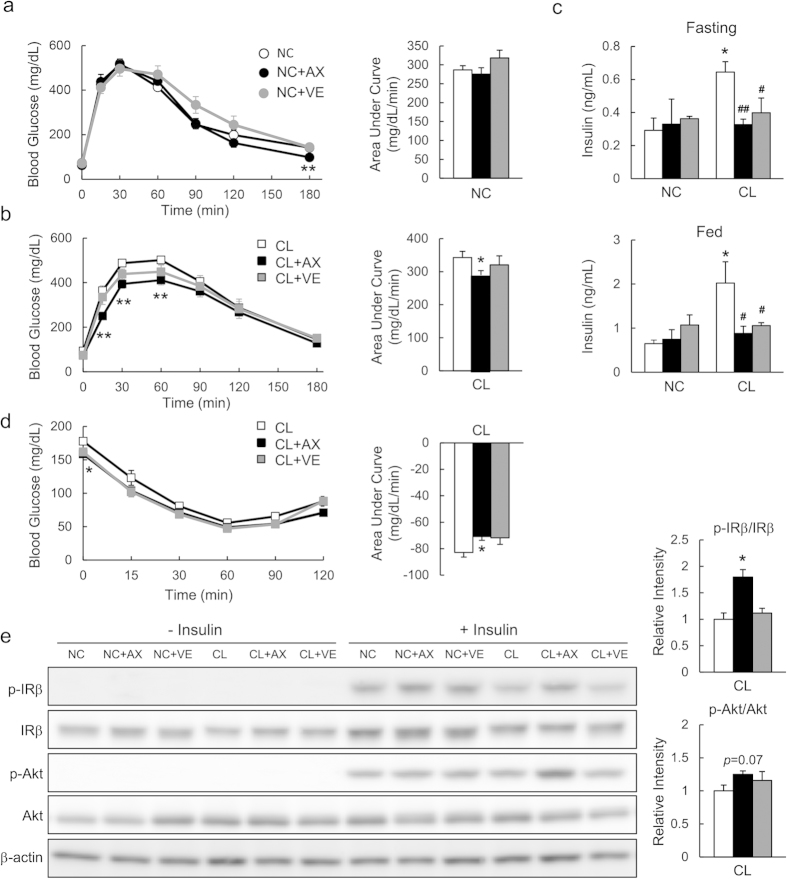
Astaxanthin ameliorated diet-induced glucose intolerance and hepatic insulin resistance. (**a**,**b**) Glucose tolerance tests (GTTs; *n* = 5–8). *P < 0.05, **P < 0.01 NC+AX group vs. NC group or CL+AX group vs. CL group. (**c**) Plasma insulin levels (*n* = 5–8). *P < 0.05 vs. mice fed a NC diet; ^#^*P* < 0.05, ^##^*P* < 0.01 vs. mice fed a CL diet. (**d**) Insulin tolerance tests (ITTs) in CL-diet fed mice (*n* = 8). *P < 0.05 vs. CL group. (**e**) Hepatic insulin signaling (*n* = 4). *P < 0.05 vs. CL group.

To determine whether astaxanthin affected glucose tolerance or insulin resistance in NASH mice, glucose tolerance tests (GTTs) and insulin tolerance tests (ITTs) were performed (Fig. 3). GTTs indicated that the administration of astaxanthin decreased blood glucose levels at 180 min in NC-fed mice, whereas vitamin E had no effect (Fig. 3a). However, CL diet-induced glucose intolerance and hyperinsulinemia in both the fasting and fed states were suppressed significantly by astaxanthin (Fig. 3b,c). Vitamin E treatment also reduced plasma insulin levels. ITTs demonstrated that CL+AX mice had slightly increased insulin sensitivity compared with CL mice (Fig. 3d). These results were associated with enhanced insulin-stimulated phosphorylation of the insulin receptor (IR)-β subunit (p-IRβ), and Akt (p-Akt) in the livers of CL+AX mice compared with CL mice, whereas vitamin E had little effect on hepatic insulin signaling (Fig. 3e). Furthermore, insulin signaling was enhanced by astaxanthin in palmitic-acid-loaded primary hepatocytes (Fig. S5a). At the cellular level, palmitic-acid-induced insulin resistance was associated with a pro-inflammatory response, such as increased phosphorylation of p38 MAPK, NF-κB p65 and ERK. These pro-inflammatory signals were slightly decreased by astaxanthin treatment (Fig. S5b). Therefore, astaxanthin protected mice against diet-induced hepatic insulin resistance and glucose intolerance.

### Astaxanthin Reduced the Activation of Both Kupffer and Stellate Cells and Attenuated Hepatic Inflammation and Fibrosis

**Figure 4:**
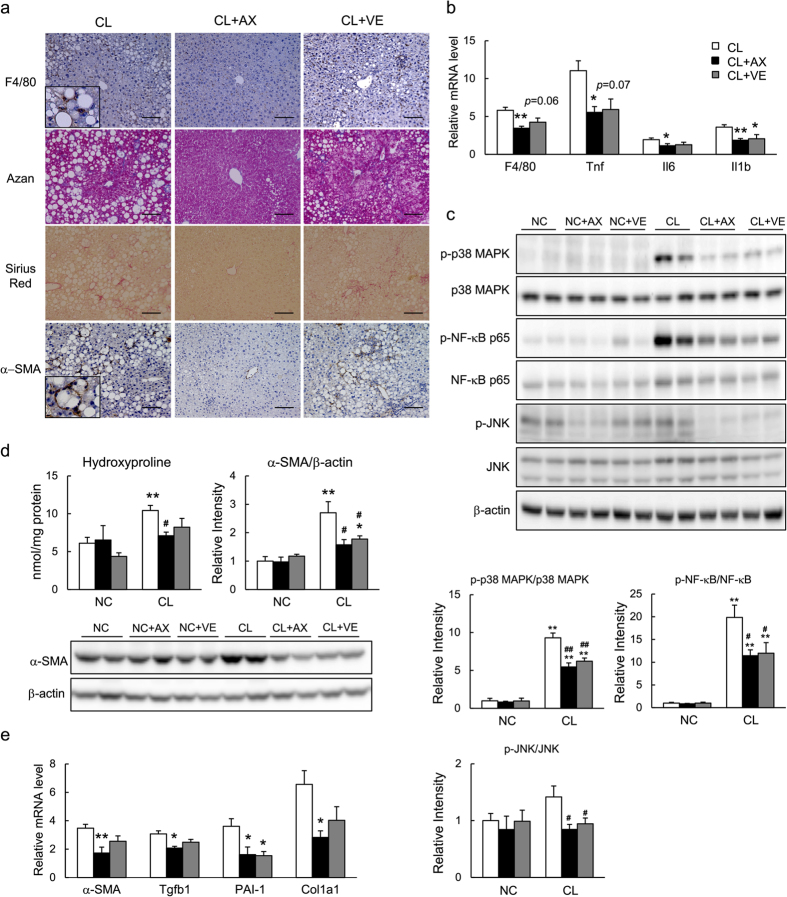
Astaxanthin attenuated hepatic inflammation and fibrosis in NASH mice. (**a**) F4/80 immunostaining, Azan and Sirius Red staining, α-SMA immunostaining; scale bars = 100 μm. (**b**) mRNA expression of *F4/80* and inflammatory cytokines in mouse livers. (**c**) Immunoblots and quantification of p-p38MAPK, p-JNK, and p-NF-κB p65 levels in the liver. (**d**) Hydroxyproline content and immunoblotting and quantification of α-SMA expression in mouse livers. (**e**) mRNA expression of fibrogenic genes in the livers. *n* = 5–8, *P < 0.05, **P < 0.01 vs. NC or CL group; ^#^*P* < 0.05, ^##^*P* < 0.01 vs. the CL group.

We confirmed previously that the number of F4/80^+^ macrophages/Kupffer cells was increased significantly in the livers of CL mice, suggesting that the CL diet induced intense inflammation in the liver^5^. Astaxanthin and vitamin E treatment markedly and slightly reduced the number of F4/80^+^ cells, respectively, as assessed by immunostaining and the analysis of mRNA expression (Fig. 4a,b). In addition, astaxanthin decreased the expression of proinflammatory cytokines, including *Tnf*, *Il6*, and *Il1b*, which were upregulated by the CL diet, to extents greater than did vitamin E (Fig. 4b, Fig. S4b). These findings were also associated with the attenuated phosphorylation of JNK, p38 MAPK, and NF-κB p65 (Fig. 4c). Therefore, astaxanthin reduced the infiltration and activation of Kupffer cells to attenuate hepatic inflammation in NASH mice.

Histological analyses using Azan and Sirius Red staining revealed that the CL diet alone induced fibrosis, as described previously^5^. Astaxanthin prevented the development of hepatic fibrosis (Fig. 4a). In addition, hydroxyproline content, a biochemical marker of hepatic collagen content, increased significantly in CL mice vs. NC mice. Importantly, astaxanthin lowered the hydroxyproline content significantly, whereas vitamin E had only a small effect (Fig. 4d). Immunohistochemical staining for α-SMA showed that the CL diet-induced increase in α-SMA-positive HSC numbers was markedly decreased by astaxanthin (Fig. 4a), and slightly by vitamin E; these observations were confirmed by immunoblotting and qPCR (Fig. 4d,e). In addition, astaxanthin inhibited the increased expression of the fibrogenic genes *Tgfb1*, *Col1a1*, and *PAI-1* caused by consumption of the CL diet, whereas vitamin E suppressed *PAI-1* mRNA expression (Fig. 4e, Fig. S4c). Combined, these results suggest that astaxanthin decreased the accumulation of collagen by inhibiting the activation of HSCs in the liver, thereby attenuating hepatic fibrosis.

### Reciprocal Decrease in M1-type Macrophages and Increase in M2-type Macrophages in the Livers of Astaxanthin-fed Mice

**Figure 5:**
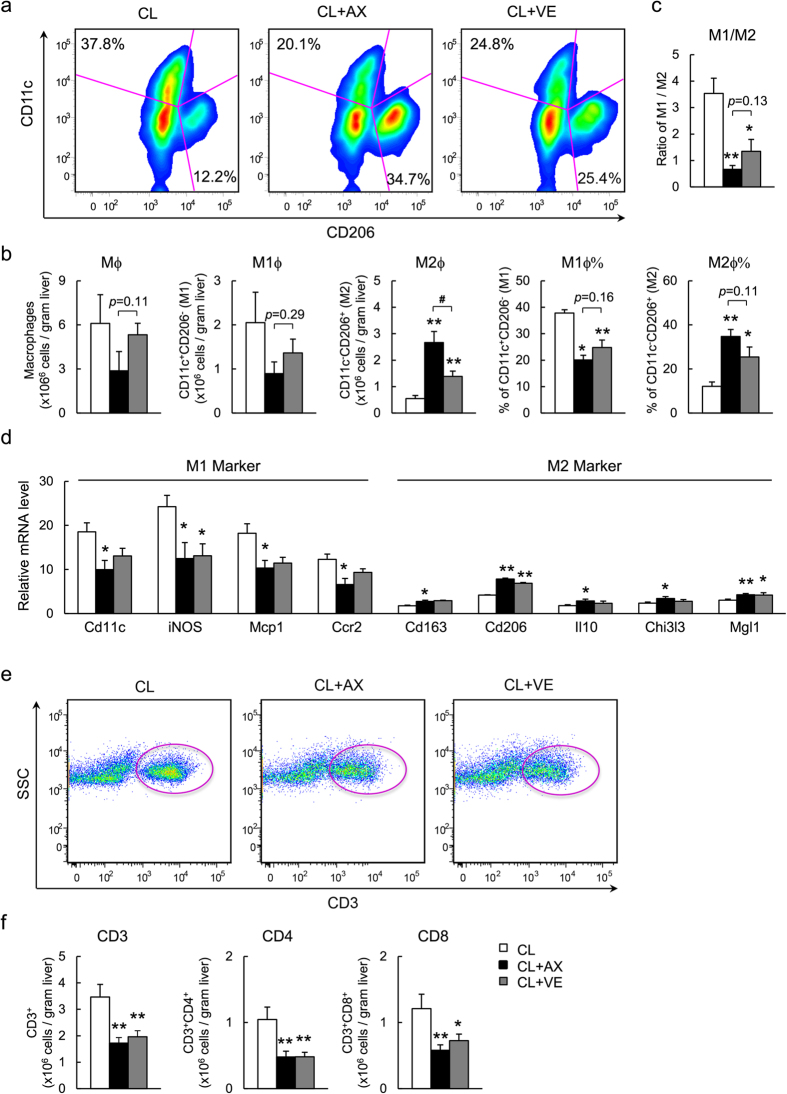
Decreased M1-type and increased M2-type macrophages in NASH livers after astaxanthin administration. (**a**,**b**) A representative plot and quantitation of M1/M2 macrophages in the livers of mice. (**c**) M1/M2 ratios. (**d**) mRNA expression of M1 and M2 macrophage markers in the livers. (**e**,**f**) A representative plot of CD3^+^ T cells and quantitation of CD3^+^, CD8^+^, CD4^+^ T cells in the livers of mice (*n* = 8). *P < 0.05, **P < 0.01 vs. the CL group, ^#^*P* < 0.05, CL+AX group vs. the CL+VE group.

To further quantify hepatic macrophage subsets, FACS was used to analyze macrophages/Kupffer cells isolated from mice (Fig. S6). Consistent with the results of immunohistochemistry, the total number of hepatic macrophages increased by 1.9-fold in mice fed the CL diet compared with the NC diet (Fig. S7a and S7b). However, CL+AX mice exhibited a slightly decreased total macrophage content compared with CL and CL+VE mice (Fig. 5a,b). Specifically, CL+AX and CL+VE mice exhibited a 56% and 33% reduced CD11c^+^ CD206^−^ (M1-type) macrophage count, respectively, whereas the number of CD11c^−^ CD206^+^ (M2-type) macrophages was increased by 3.7- and 1.5-fold, respectively. In addition, the percentage of M1-type and M2-type macrophages was decreased and increased significantly, respectively, by both astaxanthin and vitamin E treatment (Fig. 5b). These effects resulted in a predominance of M2 rather than M1 macrophage population in the livers of both astaxanthin- and vitamin E-fed mice (Fig. 5c). These results were associated with a reduction in the expression of M1 macrophage markers (*Cd11c*, *iNOS*, *Mcp1*, and *Ccr2*) and an increase in the expression of M2 macrophage markers (*Cd163*, *Cd206*, *Il10*, *Chi3l3*, and *Mgl1*) mRNA expression (Fig. 5d, Fig. S4d). However, a predominance of a Ly6C^−^ over Ly6C^hi^ monocyte population was not observed in either the peripheral blood or bone marrow of CL+AX and CL+VE mice (Fig. S7c and S7d). This suggests that astaxanthin and vitamin E caused a dynamic shift to an M2-dominant macrophage phenotype in the livers of NASH mice.

Since T cells are involved in the pathogenesis of NASH, we next assessed the effects of astaxanthin and vitamin E on T cell recruitment. The total number of CD3^+^, CD4^+^, and CD8^+^ T cells in the liver was increased significantly by CL diet feeding (Fig. S7e), and decreased by either astaxanthin (by 50%, 54%, and 52%, respectively) or vitamin E (43%, 54%, and 40%) (all *P* < 0.05; Fig. 5e,f). Therefore, astaxanthin and vitamin E suppressed the accumulation of helper and cytotoxic T cells. In addition, astaxanthin (25–100 μM) decreased the expression of LPS-induced M1 markers (*Tnf*, *Il1b*, and *Ccl5*) in RAW264.7 macrophages, but augmented IL-4-induced M2 marker expression (*Il10*, *Cd209a*, and *Chi3l3*) in a dose-dependent manner (Fig. S8a and S8b). This suggests that astaxanthin improved hepatic insulin resistance and inflammation via an M2-dominant shift in macrophages/Kupffer cells and a subsequent reduction in T cell accumulation in NASH.

### Astaxanthin Reversed Advanced NASH More Potently than Vitamin E in Mice

**Figure 6:**
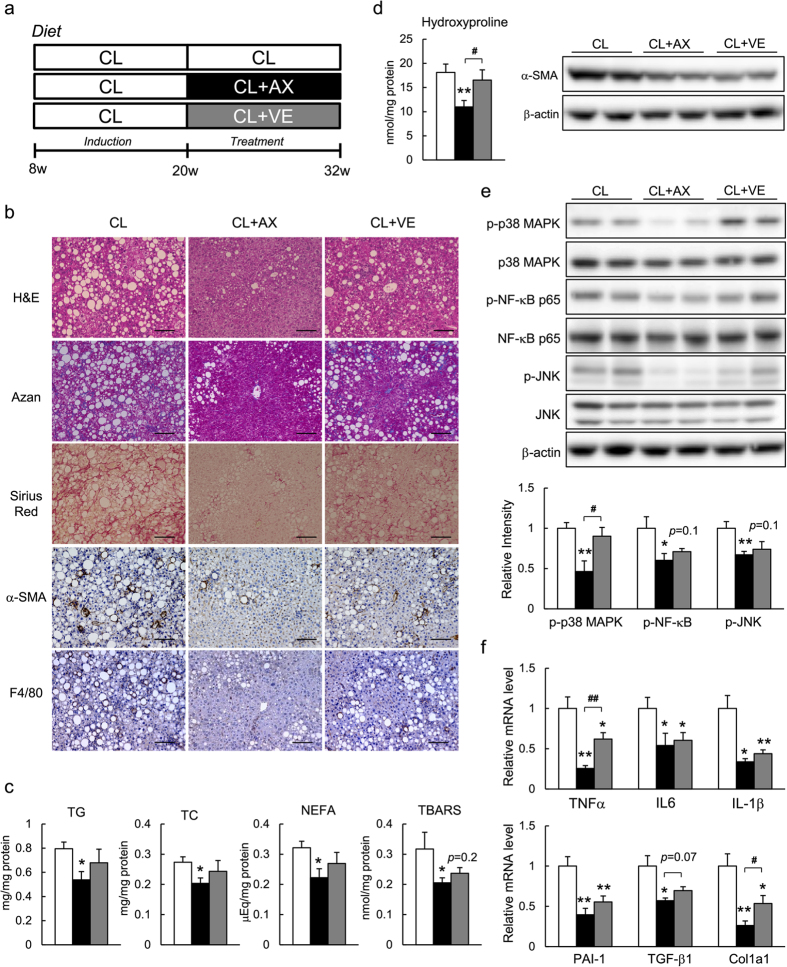
Astaxanthin reversed advanced NASH in mice. (**a**) Study design to assess the therapeutic effects of astaxanthin and vitamin E. (**b**) Histological analysis of liver sections; scale bars = 100 μm. (**c**) Hepatic TG, TC, NEFA, and TBARS levels. (**d**) Hydroxyproline content (left) and immunoblotting for α-SMA (right) in the livers. (**e**) Immunoblotting and quantification of p-p38MAPK, p-JNK, and p-NF-κB p65 levels in the livers. (**f**) mRNA expression of inflammatory cytokine and fibrogenic genes in the livers (*n* = 8). *P < 0.05, **P < 0.01 vs. the CL group; ^#^*P* < 0.05, CL+AX group vs. CL+VE group.

Next, we compared the therapeutic effects of astaxanthin and vitamin E on advanced-stage NASH in mice. After NASH was induced by feeding CL diet for 12 weeks, the CL diet with or without either astaxanthin or vitamin E was administered for an additional 12 weeks (Fig. 6a). Treatment with astaxanthin decreased plasma TG, TC, NEFA, AST, ALT and insulin levels in CL mice significantly without affecting body and liver weight (Table S1), whereas vitamin E treatment had little effect on these metabolic parameters. Astaxanthin significantly improved glucose intolerance and insulin resistance, whereas Vitamin E was less effective (Table S1, Fig. S9a and S9b). Histologically, astaxanthin treatment markedly ameliorated the macrovascular steatosis, macrophage/Kupffer cell infiltration, and fibrosis associated with HSC activation, but Vitamin E only minimally affected these histological changes (Fig. 6b,d). Potent hepatic inflammation, as characterized by increased stress or inflammatory signaling and upregulated inflammatory genes, was attenuated significantly by astaxanthin (Fig. 6e,f). Astaxanthin suppressed fibrogenic gene expression markedly, whereas vitamin E decreased hepatic inflammation and fibrogenesis only slightly (Fig. 6f). Together, these results suggest that astaxanthin was more effective at reversing advanced NASH than was vitamin E.

### Astaxanthin Alleviated NASH in Humans

**Figure 7:**
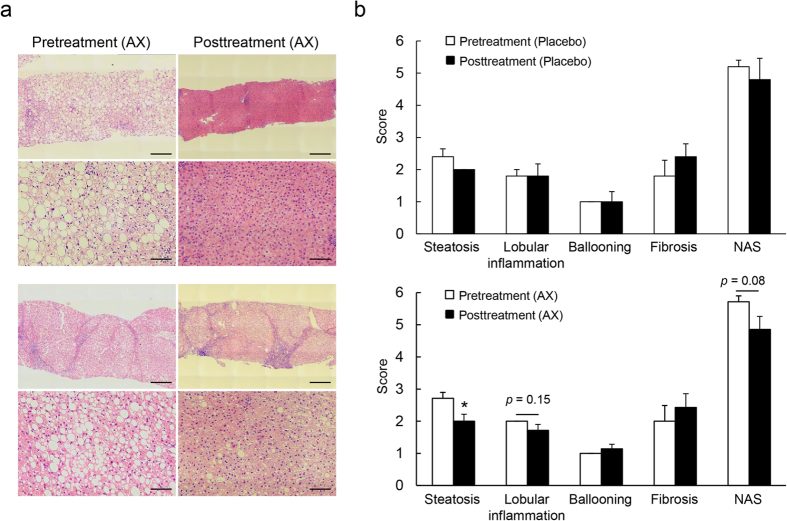
Astaxanthin alleviated NASH in humans. (**a**) Representative H&E-stained liver sections from two subjects with NASH before and after treatment revealed that astaxanthin improved steatohepatitis after 24 weeks of treatment. Scale bars = 400 μm for low magnification (upper), and 100 μm for high magnification (lower). (**b**) NAFLD activity score (NAS) in NASH subjects before and after treatment with placebo (*n* = 5) or astaxanthin (AX, *n* = 7). *P < 0.05 vs. pretreatment.

Since our results revealed promising preventative and therapeutic effects of astaxanthin on NASH in mice, we next extended our studies to humans. Twelve biopsy-confirmed NASH patients were treated orally with placebo (*n* = 5) or astaxanthin (*n* = 7) for a total of 24 weeks. The clinical background and plasma parameters of patients are shown in Table S2. After 24 weeks of astaxanthin treatment, plasma parameters involved in glucose and lipid metabolism and liver functions were unaffected by astaxanthin treatment. Moreover, no significant difference was observed in the changes from baseline between the placebo- and astaxanthin-treated patients (Table S2). However, astaxanthin treatment improved hepatic steatosis markedly in NASH patients (Fig. 7a). In addition, placebo treatment did not affect NAFLD activity score, while astaxanthin treatment reduced the grade of steatosis and tended to alleviate lobular inflammation, but did not alter the presence of ballooning or the stage of fibrosis (Fig. 7b). Together, these results suggest that astaxanthin reduces the total NAS score and alleviates human NASH.

## Discussion

This study compared the effects of the potent antioxidant carotenoid astaxanthin and vitamin E on NASH and elucidated the potential mechanism underlying the effects. We found that astaxanthin exhibited significant preventative and therapeutic effects on NASH in a lipotoxic model. Astaxanthin attenuated insulin resistance and excessive hepatic lipid accumulation and peroxidation, an increased proportion of proinflammatory or M1-type macrophages/Kupffer cells, stellate cell activation, and fibrosis in diet-induced NASH. In addition, astaxanthin ameliorated simple steatosis, the early stage of NAFLD, in both genetically obese (*ob/ob*) and DIO mice. Finally, we demonstrated that astaxanthin has the potential to improve NASH in humans.

The different mechanisms of action determined by comparing astaxanthin and vitamin E in NASH mice are intriguing because both of these lipophilic antioxidants suppress hepatic lipid peroxidation to an equivalent extent (Fig. 2d). Collectively, these results suggest that astaxanthin is similarly or more effective at preventing and treating NASH than is vitamin E (Table S3). First, astaxanthin was superior to vitamin E at improving steatosis by suppressing lipid accumulation (Fig. 2). Second, astaxanthin reduced inflammation and insulin resistance more potently than did vitamin E. Of note, these anti-inflammatory and insulin sensitizing effects were associated with attenuated MAPK (JNK/p38 MAPK) and NF-κB activation (Fig. 4), decreased macrophage/Kupffer cell and T cell accumulation, as well as alternative M2 macrophage activation in the liver (Fig. 5). Finally, astaxanthin prevented and reversed hepatic fibrosis to a greater extent than did vitamin E (Figs 4 and 6).

Hepatic macrophages, which consist of resident Kupffer cells and recruited bone marrow-derived macrophages, are the major cells that produce inflammatory mediators such as TNF-α and IL-1β to cause systemic insulin resistance as well as NASH^23^. Tissue macrophages are phenotypically heterogeneous: M1 or classically activated macrophages are stimulated by Toll-like receptor ligands such as LPS and interferon-gamma, whereas M2 or alternatively activated macrophages are stimulated by IL-4/IL-13^24^,^25^. The dysregulation of M1/M2 polarization is emerging as a central mechanism underlying the pathogenesis of chronic inflammation, atherosclerosis, obesity, and comorbidities such as insulin resistance and NAFLD^24^,^25^,^26^. More recently, M2 macrophages/Kupffer cells have been reported to protect against alcoholic and nonalcoholic fatty liver disease by promoting M1 macrophage/Kupffer cell apoptosis^27^. Therefore, strategies that modify macrophage polarization by restraining M1 or driving M2 activation might protect against inflammation and insulin resistance, and thereby halt the progression of NASH.

The current *in vitro* studies (Fig. S8) and FACS data suggested that astaxanthin caused a reciprocal decrease in M1 macrophages and increase in M2 macrophages to attenuate insulin resistance and inflammation in NASH. These results were associated with attenuated inflammatory signaling via JNK, as well as enhanced insulin signaling in the livers of CL mice (Figs 3e and 4c). This is consistent with a study by Han *et al.*, who demonstrated that JNK activation is required for M1 macrophage polarization during obesity-associated inflammation and insulin resistance^28^. T cells are critical regulators of macrophage polarization, and some studies have demonstrated that the infiltration of Th1 and CD8^+^ T cells precedes M1-polarized macrophage recruitment and contributes to insulin resistance in response to obesity^29^. Sutti *et al.* reported that lipid peroxidation induced the liver recruitment of CD4^+^ and CD8^+^ T cells, which in turn further stimulated a macrophage M1 response in a dietary model of NASH^30^. Therefore, astaxanthin suppressed the recruitment of T cells as well as M1 activation of macrophages to alleviate hepatic insulin resistance and the progression of NASH.

An important question is whether astaxanthin affects the M1/M2 status in bone marrow or peripheral blood given the link between monocyte subtypes and their fate as M1/M2 macrophages in NASH. However, astaxanthin did not affect Ly6C^hi^ or Ly6C^−^ monocyte subsets either in the bone marrow or peripheral blood (Fig. S7c and S7d). Instead, consistent with previous studies^31^, astaxanthin accumulated robustly in the livers of mice (Fig. S3c). Furthermore, astaxanthin induced M2 macrophages remarkably *in vivo* (Fig. 5b,c), and also augmented IL-4-induced M2 or the alternative activation of macrophages *in vitro* (Fig. S8b). These data suggest that astaxanthin caused a dynamic shift in the M2 polarization of macrophages/Kupffer cells within the liver, which contributed to the attenuation of insulin resistance and the progression of NASH.

It is important that the improvements in hepatic steatosis and dyslipidemia caused by astaxanthin were not secondary to a reduction in caloric intake, weight, or adiposity. The increased delivery of fatty acids to the liver and/or *de novo* lipogenesis, which are seen commonly in insulin resistance, results in oxidative stress. Our results showed that astaxanthin decreased the expression of lipogenic and lipid-uptake genes without affecting genes related to fatty acid oxidation in the liver of NASH mice (Fig. 2e). These effects would contribute to both the reduced lipid accumulation and amelioration of hepatic steatosis.

Lipid-induced cellular stresses can induce the innate immune response, insulin resistance, and fibrogenesis by activating Kupffer cells and HSCs in the liver^5^,^32^. Our data suggest that astaxanthin could reduce the associated increased oxidative stress, as shown by the reduced levels of TBARS, and thereby inhibit lipid peroxidation and the subsequent development of hepatic insulin resistance, inflammation, and fibrosis. 

Moreover, fatty acid-induced insulin resistance at the hepatocyte level is caused by reactive oxygen species (ROS)^33^. Astaxanthin, probably due to its strong antioxidant effect, decreased ROS generation and, therefore, improved insulin signaling. In addition, astaxanthin increases Akt phosphorylation, which is associated with decreased phosphorylation of ERK in cultured rat muscle cells^22^. Our results also demonstrated that astaxanthin attenuated p38 MAPK and ERK phosphorylation, which contributed to the improvement of hepatic insulin resistance.

To date, the molecular targets of vitamin E and carotenoids in NASH remain unclear. A recent sub-study of PIVENS demonstrated that the response of NASH to vitamin E treatment was associated with decreased hedgehog pathway activity^34^. Meanwhile, the biological actions of carotenoids are mediated in part by retinoic acid, an active form of provitamin A. Provitamin A carotenoids such as β-carotene and β-cryptoxanthin act via the retinoic acid receptor (RAR) to benefit human health^35^. By contrast, astaxanthin, which is similar to lutein and zeaxanthin, is a non-provitamin A carotenoid that cannot be converted to retinoid. However, some studies demonstrated that the actions of astaxanthin are mediated via RAR or retinoid signaling^36^,^37^. The concentrations of astaxanthin and vitamin E used in this study were identical and/or similar to those used in previous works^21^,^38^. It is noteworthy that astaxanthin exhibited better potential for inhibiting lipotoxicity-induced insulin resistance and NASH than did vitamin E. Notably, astaxanthin does not act as a prooxidant, unlike α-lipoic acid and vitamins C and E^39^. Therefore, astaxanthin might act via RAR or other nuclear receptors^7^,^40^ to exert effects on NASH in addition to its antioxidant effect. Additional studies are needed to better understand the potential of astaxanthin in NASH and investigate whether astaxanthin and vitamin E have common or unique molecular mechanisms of action. Furthermore, a combination of astaxanthin and vitamin E might exert a synergistic effect, improving the prevention or treatment of NASH.

In summary, this study demonstrated that astaxanthin, a potent antioxidant carotenoid, inhibited and reversed lipotoxicity-induced insulin resistance and steatohepatitis in mice by attenuating hepatic lipid accumulation and peroxidation. The beneficial effects of astaxanthin were attributable in part to both the decreased hepatic recruitment of T cells and macrophages, as well as an M2-dominant polarization of macrophages/Kupffer cells to attenuate whole-body insulin resistance and hepatic inflammation and fibrosis. Astaxanthin was more effective at preventing and treating NASH than was vitamin E in mice. Taken together with our preliminary results indicating improvement in human NASH, astaxanthin might be a novel and promising treatment for NASH.

## Methods

### Mice and Diets

Seven-week-old male C57BL/6J mice and 5-week-old male *ob/ob* mice were purchased from Charles River Laboratories (Yokohama, Japan). For hepatic steatosis experiments, C57BL/6J mice and *ob/ob* mice were fed normal chow (NC) that contained 10% of calories from fat (CRF-1, Charles River) or a high-fat diet (HFD) that provided 60% of calories from fat (Research Diets Inc., New Brunswick, NJ, USA) with or without 0.02% astaxanthin (Fuji Chemical Industry Co Ltd, Toyama, Japan)^22^ for 10 weeks. For NASH experiments, the mice were divided into six groups and fed for 12 weeks as follows: 1, NC; 2, NC containing 0.02% astaxanthin (NC+AX); 3, NC containing 0.02% vitamin E (NC+VE); 4, high-fat, cholesterol, and cholate diet (CL, also referred to as atherogenic high-fat; Research Diets; 60% of calories from fat, 1.25% cholesterol, 0.5% sodium cholate)^5^; 5,CL diet with 0.02% astaxanthin (CL+AX); or 6, CL diet with 0.02% vitamin E (CL+VE). All mice were maintained on a 12/12-h light/dark cycle and were given free access to food and water.

All animal procedures were performed in accordance with the standards set forth in the Guidelines for the Care and Use of Laboratory Animals at Kanazawa University, Japan. The protocols were approved by the Institute for Experimental Animals of Kanazawa University.

### Measuring Astaxanthin Concentrations

After 8-week-old C57BL/6J mice had been fed a CL or CL+AX diet for 3 weeks and then fasted for 2 h, tissues were collected and snap-frozen in liquid nitrogen. The astaxanthin concentrations in tissues were then measured using HPLC, as described previously^31^; data are reported as μg/g organ.

### Lipid, Glucose, and Insulin Determinations

Plasma triglycerides (TG), total cholesterol (TC), non-esterified fatty acids (NEFA), AST, ALT, glucose, and insulin levels, and hepatic TG, TC, and NEFA concentrations were measured as described previously^5^,^41^. Liver thiobarbituric acid reactive substrates (TBARS) were extracted and measured according to the instructions provided with the TBARS assay kit (Cayman Chemicals, Ann Arbor, MI). All hepatic lipid levels were normalized to liver protein levels.

Glucose tolerance tests (GTTs) was conducted after an overnight fast after 12 weeks of feeding. After baseline blood collection, mice were injected intraperitoneally with 2 g/kg glucose. One week later, insulin tolerance tests (ITTs) were performed after a 4-h fast and mice were injected intraperitoneally with 0.5 U/kg human insulin.

### Histological Examination and Immunohistochemistry

Paraffin-wax-embedded liver sections were stained with H&E, Azan, and Sirius Red, and immunohistochemistry for F4/80 or α-SMA was performed as described previously^26^,^41^.

### Hydroxyproline Assay

To assess liver collagen content, hydroxyproline levels were measured using a spectrophotometric assay, as described previously^42^. Briefly, liver tissue was homogenized in 1-mL ice-cold saline, and the homogenates were incubated on ice for 30 min with 125 μL of 50% trichloroacetic acid. Subsequently, the precipitated pellets were hydrolyzed for 24 h at 110 °C in 6 N HCl, filtered, and neutralized with 10 N NaOH. The resulting hydrolysates were then oxidized with chloramine-T (Sigma-Aldrich) for 25 min. The reaction mixture was then incubated in Ehrlich’s perchloric acid solution at 65 °C for 20 min. Finally, the absorbance at 560 nm was measured after samples had been cooled to room temperature. The hydroxyproline content was normalized to liver protein levels.

### Quantitative Real-Time PCR

Total RNA was isolated from frozen liver samples using a GenElute Mammalian Total RNA Miniprep Kit (Sigma-Aldrich). cDNA was synthesized using a High-Capacity cDNA Reverse Transcription Kit (Applied Biosystems, Carlsbad, CA, USA). Quantitative real-time PCR (qPCR) was then performed on a CFX384 machine (Bio-Rad, Hercules, CA, USA) using SYBR Green Master Mix, as described previously^26^. The primers used for real-time PCR are shown in Table S4. The mRNA expression levels in the groups were normalized to those of NC-fed mice.

### Immunoblots

Tissues were homogenized and sonicated in radioimmunoprecipitation assay (RIPA) lysis buffer (Millipore, Billerica, MA, USA), supplemented with protease and phosphatase inhibitors (Roche Diagnostics). The primary antibodies used were anti-phospho- c-Jun N-terminal kinase (JNK) (Thr183/Tyr185; #9255), anti-JNK (#9258), anti-phospho-p38 mitogen-activated protein kinases (p38 MAPK) (Thr180/Tyr182; #9211), anti-p38 MAPK (#9212), anti-phospho-p44/42 MAPK (ERK1/2) (Thr202/Thr204; #9101), anti-ERK1/2 (#9102), anti-phospho-nuclear factor kappa light chain-enhancer of activated B cells (NF-κB) p65 (Ser536; #3033), anti-NF-κB (#3034), anti-phospho-c-Jun (Ser73; #3270), anti-c-Jun (#9165), anti-phospho-insulin receptor β subunit (IR-β) (Tyr1146; #3021), anti-IR β (#3025), anti-phospho-Ser473 serine/threonine protein kinase Akt (Akt; #9271), anti-Akt (#9272), caspase-3 (#14220) (all from Cell Signaling Technology [Danvers, MA]), anti-α-SMA (sc-53141) (Santa Cruz Biotechnology [Santa Cruz, CA]), and monoclonal anti β-actin (A5441) (Sigma-Aldrich).

### Fluorescence-activated Cell Sorting (FACS) Analysis

The left lobes of the livers were lysed gently and digested for 20 min at 37 °C using type IV collagenase (Sigma-Aldrich, St. Louis, MO, USA) and type I deoxyribonuclease in PBS containing 2% BSA (pH 7.4). Non-parenchymal cells were incubated with Fc Block™ (BD Biosciences, San Jose, CA, USA) and then incubated with fluorochrome-conjugated antibodies (Table S5). Cells were analyzed using FACSAria II (BD Bioscience) as described previously^26^. Data analysis and compensation were performed with the aid of FlowJo (Tree Star, Ashland, OR, USA).

### Isolation of Primary Hepatocytes and Cell Culture Experiments

Mouse primary hepatocytes were isolated from a male C57BL/6J mouse (8–12 weeks) as described previously^43^. After growth in Dulbecco’s modified Eagle’s medium (DMEM, [Gibco, Invitrogen, Carlsbad, CA]) without fetal bovine serum (FBS) for 6 h, the cells were treated with 400 μM oleic acid (Sigma-Aldrich) and astaxanthin (25, 50, 100 μM) for 16 h and then harvested. To measure the hepatocyte lipid content, cells were fixed in 10% formalin for 1 h, washed with 60% isopropanol, and stained with Oil Red O (Sigma-Aldrich) for 10 min. The cells were then washed repeatedly with water, photographed, and destained using 100% isopropanol for 15 min. The optical density of the isopropanol solution was measured at 500 nm. Cellular TG levels were measured as described previously^32^. To examine the effect of astaxanthin on apoptosis and cell viability, the cells were serum-starved for 6 h, and then were treated with 400 μM palmitic acid (Sigma-Aldrich) and astaxanthin (25, 50, 100 μM) for 16 h. Cell Counting Kit-8 (CCK-8) assay (Dojindo Laboratories, Kumamoto, Japan) was performed to measure cell viability according to the manufacturer’s instructions. To assess insulin signaling, the hepatocytes were pretreated with 400 μM PA and 100 μM astaxanthin for 16 h, followed by stimulation with 100 nM insulin (Sigma-Aldrich) for 10 min. Mouse RAW264.7 macrophages were grown in DMEM supplemented with 10% FBS in a humidified atmosphere of 5% CO_2_ at 37 °C until the cells reached 90% confluence. The cells were then serum-starved for 6 h, and co-incubated with 1 μg/mL lipopolysaccharide (LPS) (Sigma-Aldrich) or 10 ng/mL IL-4 (Sigma-Aldrich) and 25, 50, or 100 μM astaxanthin for 24 h.

### Human Liver Samples

Paraffin-embedded human liver tissues were obtained from liver biopsy samples of patients with NAFLD in a randomized clinical trial performed at Kanazawa University Hospital, Japan (UMIN000008524) from March 2012 to September 2013. Patients with serum ALT level over 31 IU/L, and alcohol intake less than 30 g/day for male, and 20 g/day for female were participated in this trial, and orally administrated with 12 mg/day for total 24 weeks. Liver biopsies were conducted before and end of the trial, and read by two hepatopathologist who was blinded to clinical data. Liver biopsies were scored using the NASH Clinical Research Network Histologic Scoring System^44^. The NAFLD activity score (NAS) was calculated as an unweighted sum of the grade of steatosis (0–3), grade of lobular inflammation (0–3), and presence of ballooning (0–2). Progressed NASH was regarded as fibrosis stages 2–4, whereas early NASH was defined as fibrosis stages 0–1. NASH patients were diagnosed histologically with an NAFLD activity score (NAS) of ≥5 were analyzed in this study. The study was approved by the ethics committee of Kanazawa University Hospital, and the methods were carried out in accordance with the approved guidelines. Informed consent was collected from each participant according to the protocol approved by Kanazawa University Hospital.

### Statistical Analysis

All data are presented as means ± SEM. Differences between the mean values from two groups were assessed using two-tailed Student’s *t*-tests. Differences in mean values among more than two groups were determined using ANOVA. *P* values < 0.05 were considered to indicate statistical significance.

## Additional Information

**How to cite this article**: Ni, Y. *et al.* Astaxanthin prevents and reverses diet-induced insulin resistance and steatohepatitis in mice: A comparison with vitamin E. *Sci. Rep.* **5**, 17192; doi: 10.1038/srep17192 (2015).

## Supplementary Material

Supplementary Information

## Figures and Tables

**Table 1:** Effects of astaxanthin (AX) and vitamin E (VE) on metabolic parameters after 12 weeks of treatment.

	NC	NC+AX	NC+VE	CL	CL+AX	CL+VE
Body weight (g)	32.2 ± 0.6	33.4 ± 0.6	31.6 ± 0.8	34.0 ± 1.0	33.1 ± 0.6	34.6 ± 0.7
Food intake (g/day/kg BW)	86.9 ± 4.7	89.5 ± 4.9	89.4 ± 3.6	75.6 ± 1.7	80.0 ± 1.5	75.7 ± 3.2
Liver weight ratio (%)	3.6 ± 0.3	3.8 ± 0.3	3.9 ± 0.1	5.0 ± 0.1**	4.8 ± 0.1**	4.9 ± 0.1**
Plasma TG (mg/dL)	96.0 ± 4.5	86.8 ± 4.0	111.3 ± 6.3	43.4 ± 3.1**	32.0 ± 2.8**^#^	33.8 ± 4.8**
Plasma TC (mg/dL)	96.9 ± 3.8	92.3 ± 2.6	93.1 ± 5.0	154.2 ± 3.8**	122.2 ± 4.5**^#^	137.3 ± 4.1**
Plasma NEFA (mEq/L)	1.34 ± 0.13	1.23 ± 0.10	1.29 ± 0.12	0.98 ± 0.07	0.62 ± 0.09**^##^	0.92 ± 0.05*
Plasma AST (IU/L)	16.7 ± 5.6	13.6 ± 1.1	18.4 ± 3.3	43.5 ± 2.3**	31.6 ± 2.9**^#^	43.7 ± 0.4**
Plasma ALT (IU/L)	5.5 ± 1.3	4.8 ± 0.4	5.4 ± 1.8	18.8 ± 1.5**	10.8 ± 0.8**^##^	17.3 ± 0.4**

Data were obtained from 20-week-old fasted mice that had been fed different diets. Data are presented as means ± SEM (*n* = 5 for the NC, NC + AX, and NC + VE groups, and *n* = 8 for the CL, CL + AX, and CL + VE groups). *P < 0.05, **P < 001 vs. mice fed normal chow (NC); ^#^*P* < 0.05, ^##^*P* <  0.01 vs. mice fed CL diet.
