# Supplementary material for: Astaxanthin prevents and reverses diet-induced insulin resistance and steatohepatitis in mice: A comparison with vitamin E
Source: Sci Rep. 2015 Nov 25;5:17192. doi: 10.1038/srep17192 (PMC4658633; doi:10.1038/srep17192)
Supplement: Supplementary Information [file srep17192-s1.pdf]

Supplementary Information

Astaxanthin prevents and reverses diet-induced insulin resistance and steatohepatitis in mice: A comparison with vitamin E

Yinhua Ni,[1,2] Mayumi Nagashimada,[1] Fen Zhuge,[1] Lili Zhan,[1] Naoto Nagata,[1] Akemi Tsutsui,[3] Yasuni Nakanuma,[3] Shuichi Kaneko,[2] Tsuguhito Ota[1,2,*]

[1]Department of Cell Metabolism and Nutrition, Brain/Liver Interface Medicine Research Center, Kanazawa University, Kanazawa, Ishikawa 920-8640, Japan

[2]Department of Disease Control and Homeostasis, Kanazawa University Graduate School of Medical Science, Kanazawa, Ishikawa 920-8640, Japan

[3]Department of Pathology, Kanazawa University Graduate School of Medical Science, Kanazawa, Ishikawa 920-8640, Japan

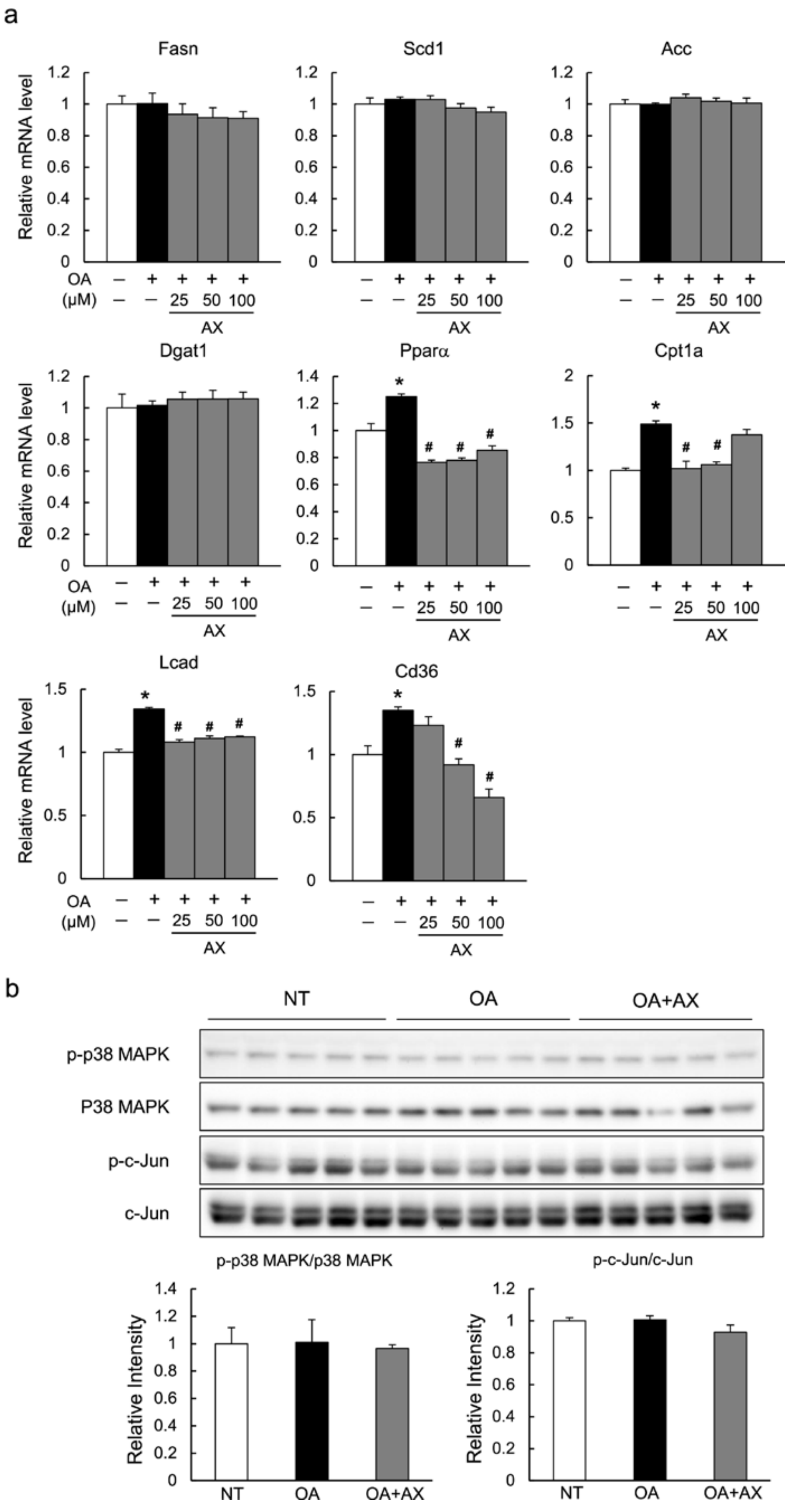
a
Fasn
Scd1
Acc
Dgat1
Pparα
Cpt1a
Lcad
Cd36
Relative mRNA level
OA
(μM)
25
50
100
AX
b
NT
OA
OA+AX
p-p38 MAPK
P38 MAPK
p-c-Jun
c-Jun
p-p38 MAPK/p38 MAPK
p-c-Jun/c-Jun
Relative Intensity

Figure S1. Effect of astaxanthin on lipid metabolism in hepatocytes. Primary hepatocytes were treated with 400 μM oleic acid (OA) and astaxanthin (25, 50, 100 μM) for 16 h. (a) mRNA expression of lipogenic and fatty acid oxidation genes in hepatocytes (n = 6). $^*P < 0.05$, vs. control incubation; $^{\#}P < 0.05$ vs. OA-treated cells. (b) Immunoblots and quantification of p-p38MAPK and p-c-Jun levels in hepatocytes treated with OA and 100 μM astaxanthin (n = 5).

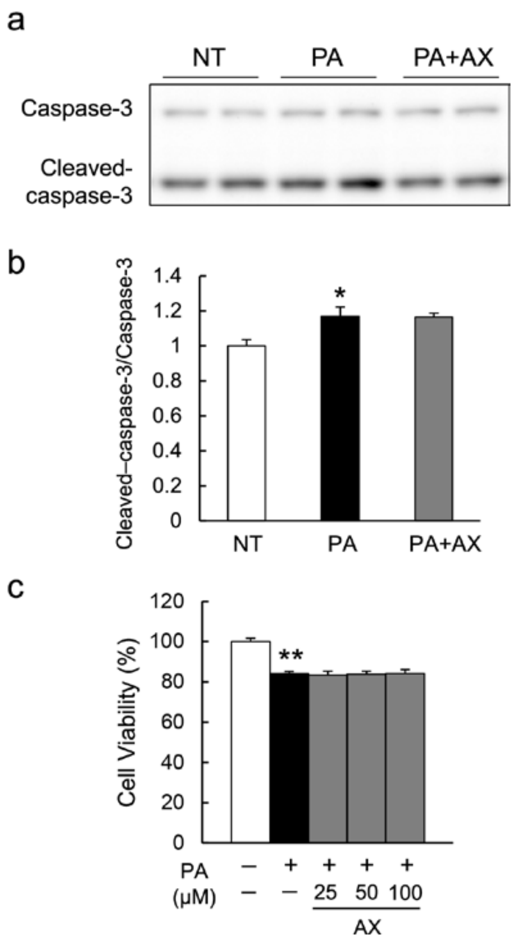

Figure S2. Astaxanthin had no effect on apoptosis and viability of hepatocytes. Primary hepatocytes were treated with 400 μM palmitic acid (PA) and astaxanthin (25, 50, 100 μM) for 16 h. (a, b) Immunoblots and quantification of cleaved-caspase-3 and caspase-3 in hepatocytes treated with PA and 100 μM astaxanthin (n = 6). *P < 0.05 vs. NT. (c) Viability of hepatocytes treated with PA and astaxanthin (n = 8). **P < 0.01 vs. control.

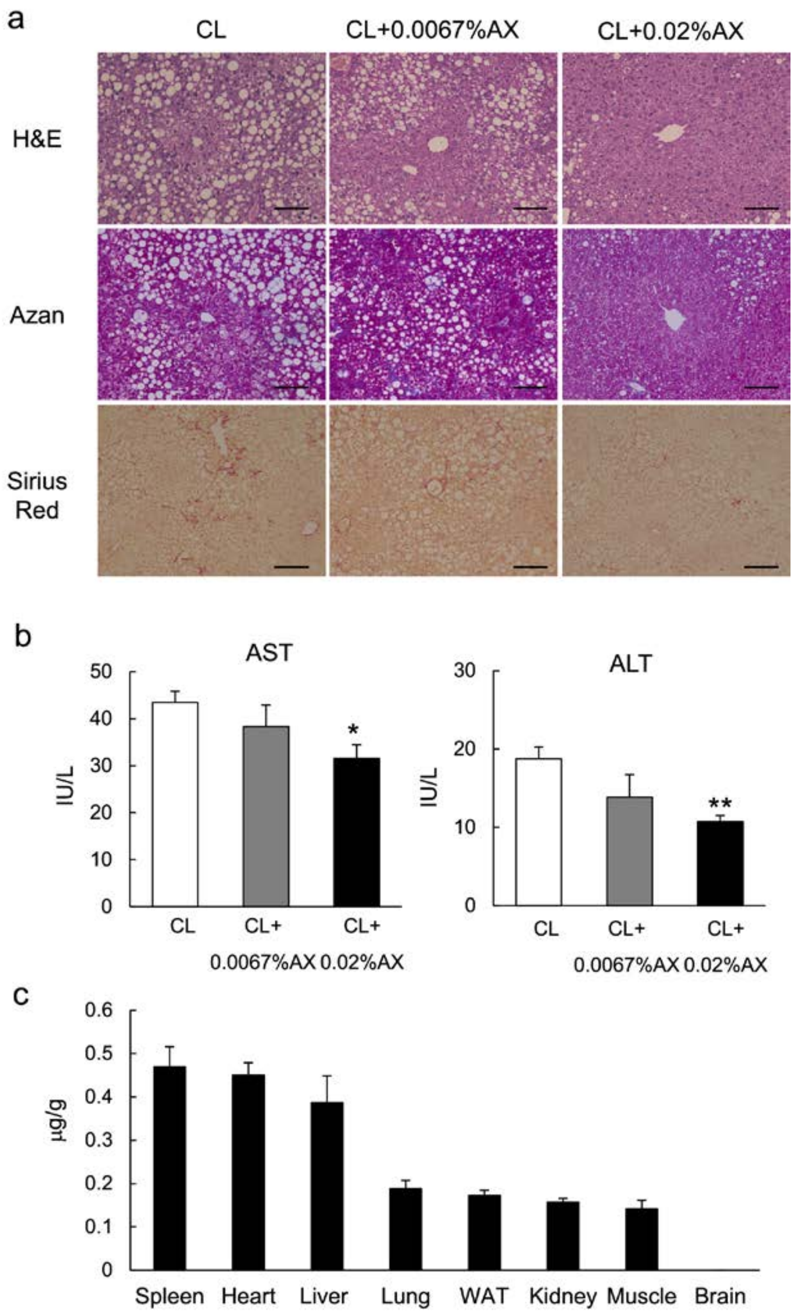

Figure S3. Dose-dependent effects of astaxanthin on the prevention of diet-induced NASH. (a) H&E, Azan, and Sirius Red-stained liver sections; scale bars = 100 μm. (b) Plasma AST and ALT levels (n = 8). *P < 0.05, **P < 0.01 vs. mice fed the CL diet. (c) The astaxanthin concentrations in various tissues were measured using HPLC (n = 5).

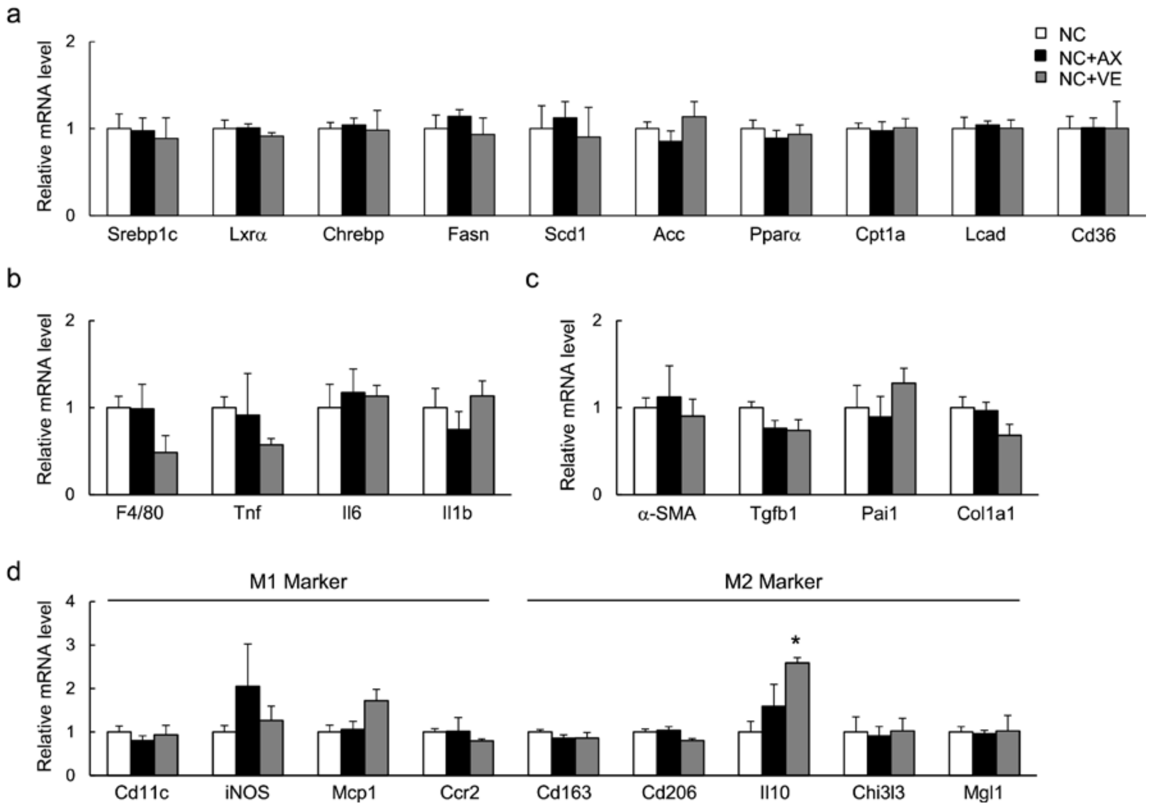

Figure S4. Astaxanthin had little effect on gene expression in the livers of NC-fed mice. (a) mRNA expression of lipogenic and fatty acid oxidation genes. (b) mRNA expression of *F4/80* and inflammatory cytokines. (c) mRNA expression of fibrogenic genes. (d) mRNA expression of M1 and M2 macrophage markers (n = 5/group). *P <0.05 vs. the NC group.

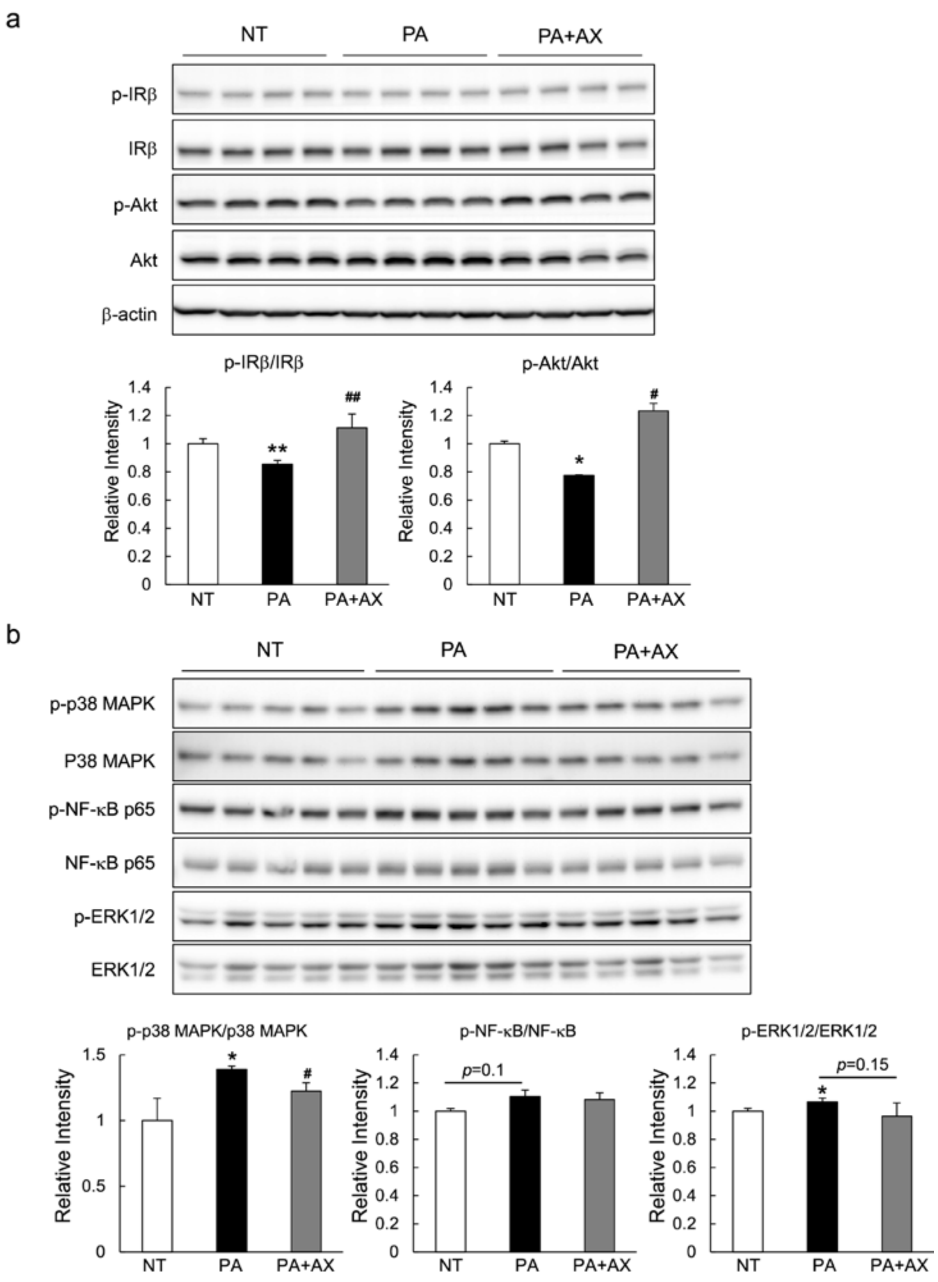

Figure S5. Astaxanthin enhanced insulin signaling in primary hepatocytes. Primary hepatocytes were pretreated with 400 µM palmitic acid (PA) and 100 µM astaxanthin for 16 h and insulin was then added for 10 min to assess insulin signaling. (a) Immunoblots and quantification of p-IRβ and p-Akt levels in hepatocytes (n = 4). (b) Immunoblots and quantification of p-p38MAPK, p-NF-κB p65 and p-ERK levels in hepatocytes (n = 5). *P < 0.05, **P < 0.01, vs. NT; $^{\#}$P < 0.05, $^{\#\#}$P < 0.01 vs. PA-treated cells.

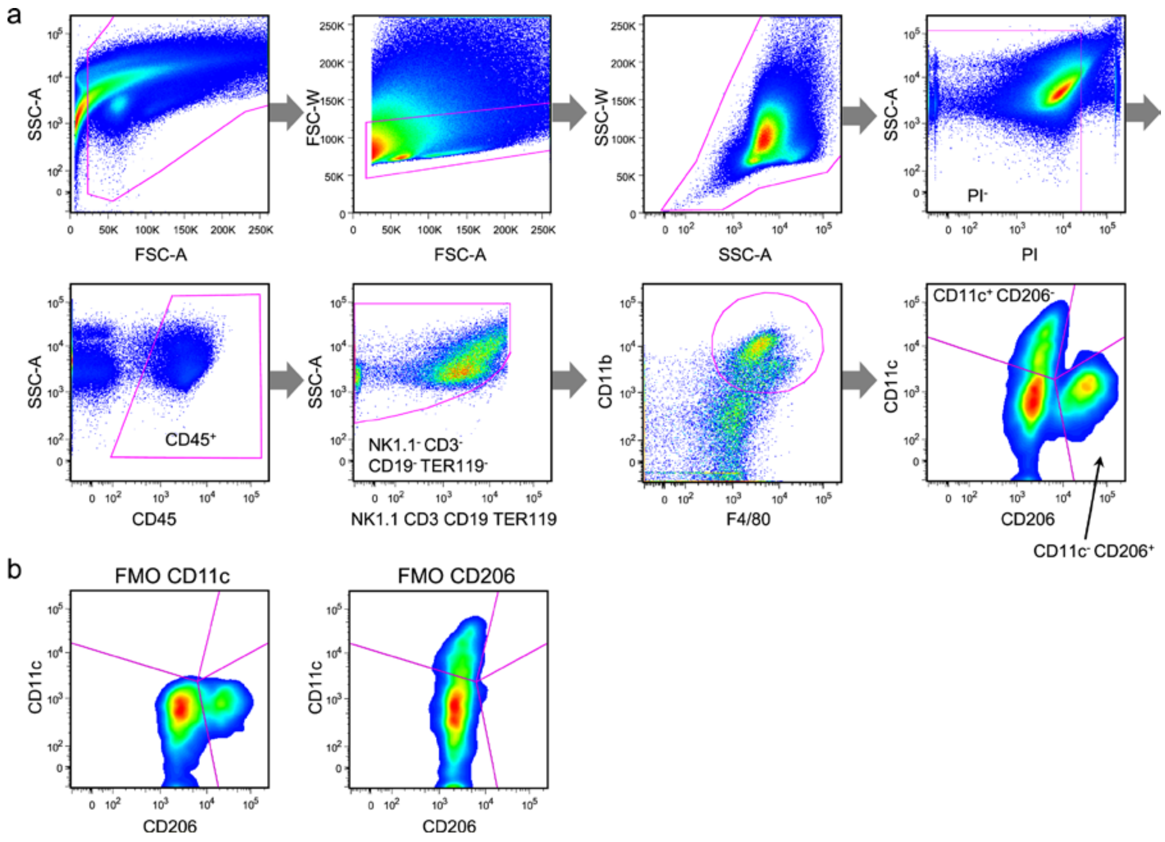

Figure S6. Sequential gating strategy for analysis of M1- and M2-type macrophages in mouse livers. (a) FACS plots of the non-parenchymal liver cell fraction isolated from mice fed the CL, CL+AX, or CL+VE diet for 12 weeks. After excluding cell debris, doublets, and dead cells using FSC/SSC parameters and propidium iodide (PI) staining, macrophages were identified as PI^{-} $CD45^{+}$ $NK1.1^{-}$ $CD3^{-}$ $CD19^{-}$ $TER119^{-}$ $CD11b^{+}$ $F4/80^{+}$ cells. M1 and M2 macrophages were gated as $CD11c^{+}$ $CD206^{-}$ and $CD11c^{-}$ $CD206^{+}$, respectively. (b) FACS plot of “fluorescence minus one” (FMO) controls for CD11c (left) and CD206 (right). FMO controls were used for gating to identify highly pure populations of M1- or M2-type macrophages.

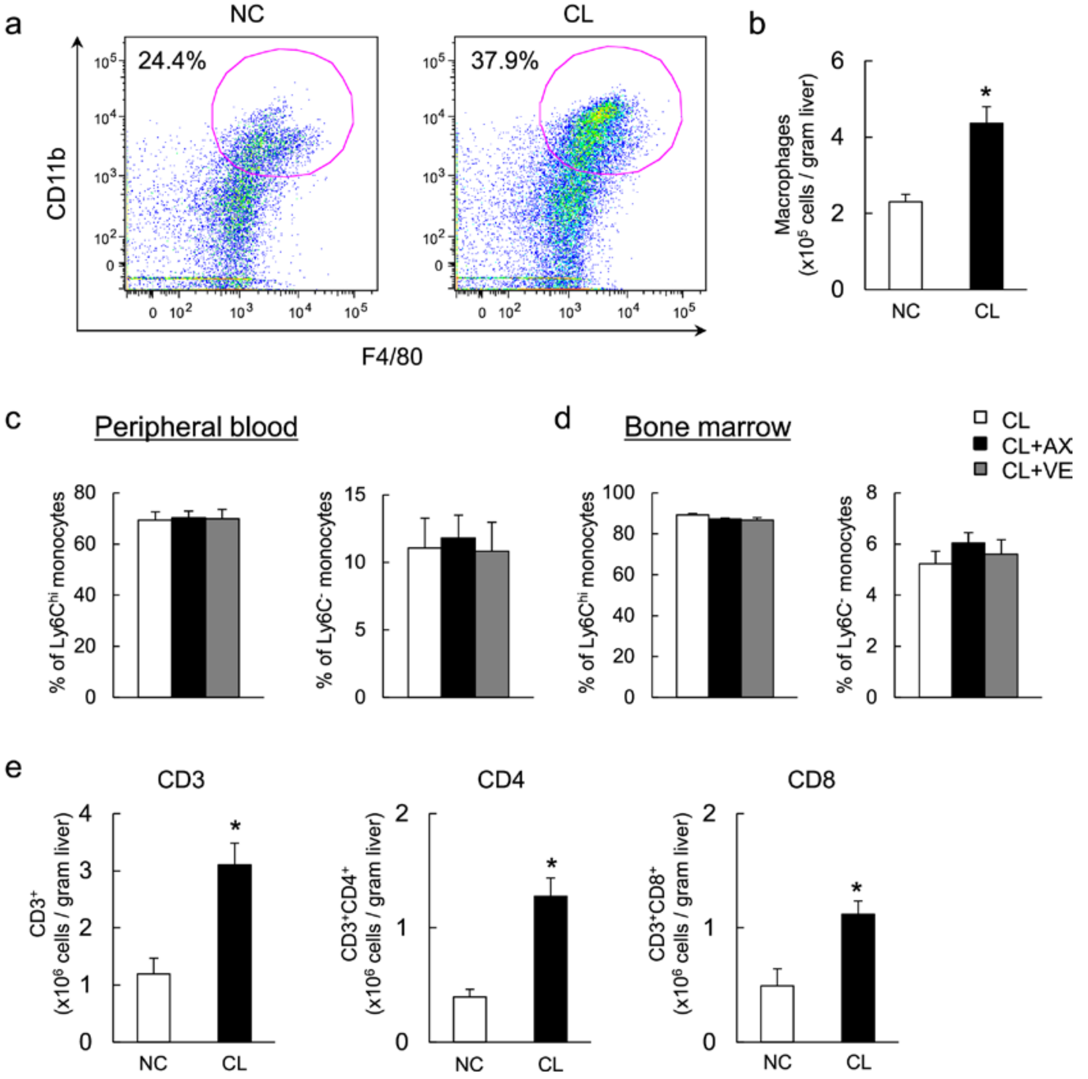

Figure S7. FACS of liver macrophages, T cells, and blood and bone marrow Ly6C^{hi} and Ly6C^{-} monocytes. (a) Representative plot demonstrating that a CL diet induced the accumulation of liver macrophages in mice. (b) Quantitation of liver macrophages in the livers of NC- or CL-diet-fed mice (n = 8). *P < 0.05 vs. mice fed the NC diet. (c, d) Percentage of Ly6C^{hi} and Ly6C^{-} monocytes in the peripheral blood and bone marrow of mice fed the CL, CL+AX, or CL+VE diet (n = 8). Monocytes were defined as CD45^{+} CD11b^{+} Gr1^{-} CD3^{-} CD19^{-} NK1.1^{-} cells. (e) Quantitation of CD3^{+}, CD4^{+}, CD8^{+} T cells in liver (n = 8). *P < 0.01 vs. mice fed the NC diet.

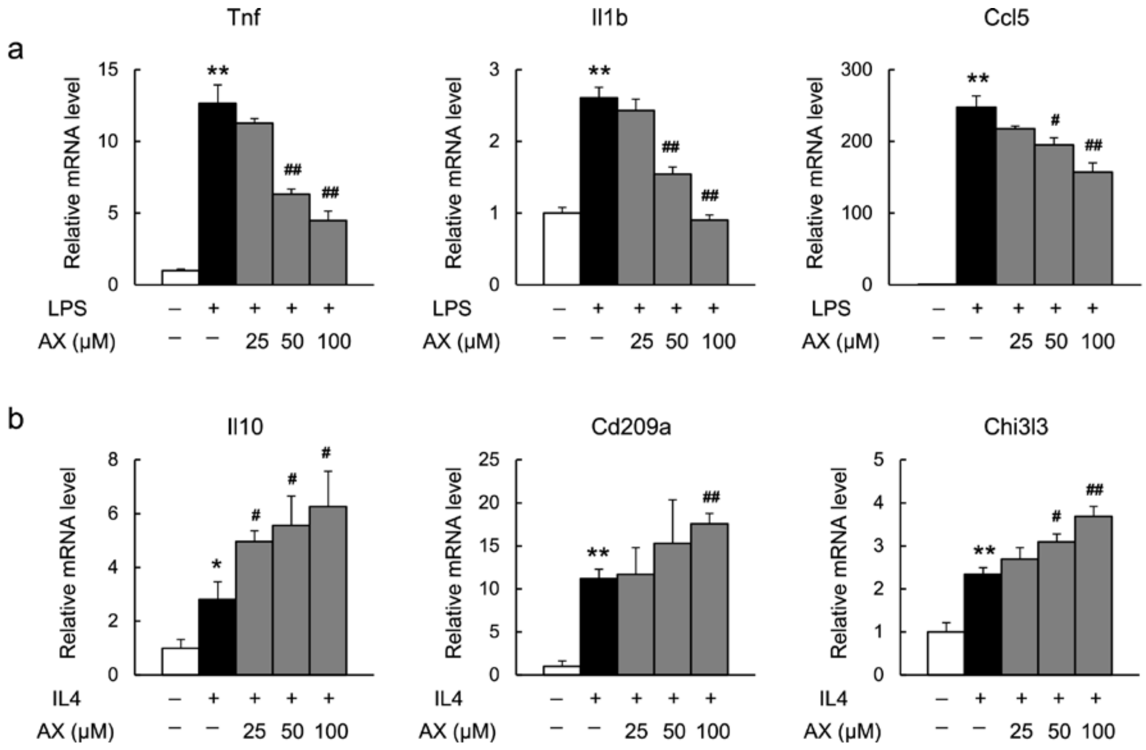

Figure S8. Astaxanthin inhibited M1 macrophage activation and enhanced M2 macrophage activation *in vitro*. Astaxanthin suppressed LPS-induced M1 marker mRNA expression (a) but augmented IL-4-induced M2 marker mRNA expression (b) *in vitro*. RAW264.7 macrophages were serum-starved for 6 h, and then co-incubated with LPS (1 μg/mL) or IL-4 (10 ng/mL) and astaxanthin (25–100 μM) for 24 h ($n = 6$). $^{*}P < 0.05$, $^{**}P < 0.01$ vs. control; $^{\#}P < 0.05$, $^{\#\#}P < 0.01$ vs. LPS- or IL-4–stimulated cells.

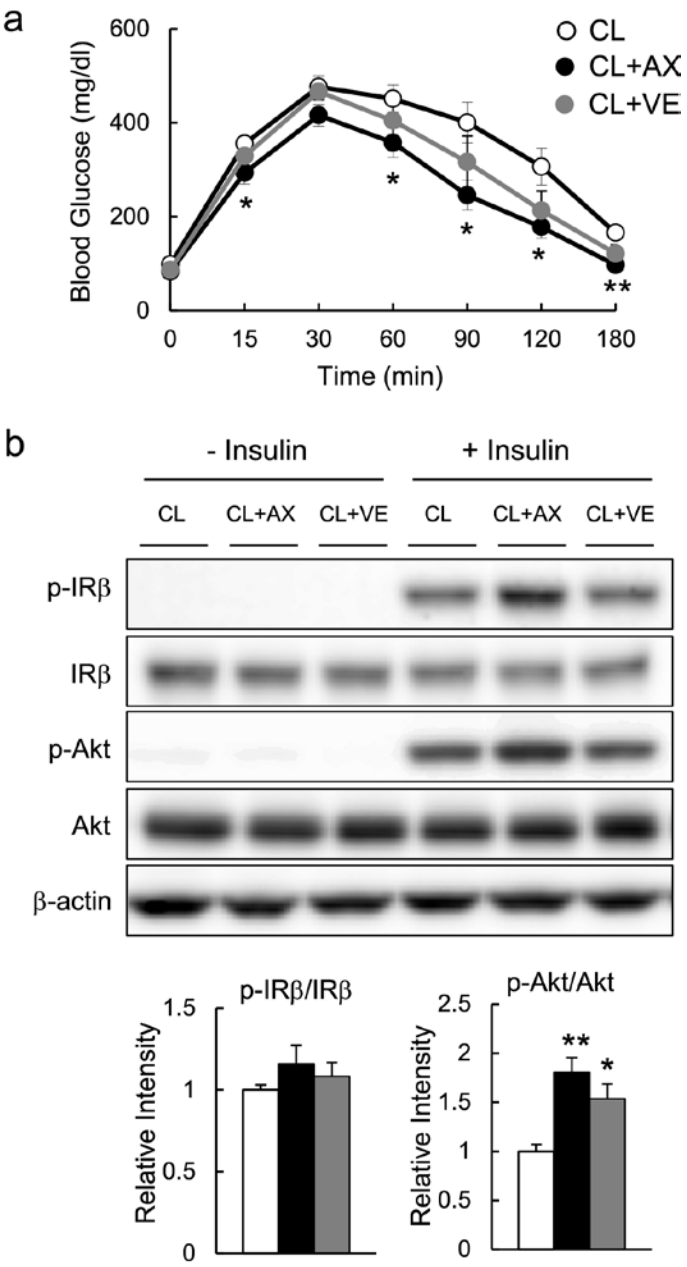

Figure S9. The therapeutic effects of astaxanthin and vitamin E in NASH. (a) GTTs in mice fed the CL, CL+AX, or CL+VE diet (n = 8). *P < 0.05, **P < 0.01 CL+AX vs. CL group. (b) Hepatic insulin signaling (n = 4). *P < 0.05, **P < 0.01 vs. CL group.

Table S1. Effects of astaxanthin (AX) and vitamin E (VE) on metabolic parameters after 12 weeks of treatment.

	CL	CL+AX	CL+VE
Body weight (g)	40.0±1.1	38.9±1.1	38.0±2.0
Liver weight ratio (%)	5.49±0.19	5.32±0.21	5.49±0.21
Plasma TG (mg/dL)	57.4±4.3	43.1±4.4*	50.4±2.5
Plasma TC (mg/dL)	173.4±4.5	149.0±3.4*	165.0±4.2
Plasma NEFA (mEq/L)	0.81±0.07	0.57±0.06*	0.66±0.10
Plasma AST (IU/L)	44.3±4.3	26.5±1.7*	37.1±3.0
Plasma ALT (IU/L)	16.8±1.3	12.1±1.2*	14.9±2.2
Plasma insulin (fasting) (ng/mL)	0.90±0.19	0.43±0.05*	0.49±0.18
Plasma insulin (fed) (ng/mL)	1.56±0.08	1.01±0.12**	1.28±0.15

Data were obtained from 32-week-old fasted mice, and are presented as means ± SEM (n = 8). *P < 0.05, **P < 0.01 vs. untreated CL-diet-fed mice.

Table S2. Plasma parameters of patients who completed the study at baseline and 6 months.

Variable	Astaxanthin (*n*=7)			Placebo (*n*=5)			
	Before	After	p^a	Before	After	p^a	p^b
Male/female	3/4			3/2			
Age (years)	50.0±6.2			58.6±6.6			
BMI (kg/m^2)	34.2±4.0	34.4±3.8	0.980	26.5±1.9	26.3±1.9	0.944	0.480
Glucose (mg/dL)	111.0±6.0	115.8±26.8	0.669	121.3±11.7	120.5±13.9	0.968	0.759
Insulin (μU/mL)	27.0±7.4	28.0±7.6	0.921	11.3±2.1	11.8±4.1	0.908	0.945
HOMA-IR	7.1±1.8	7.4±1.5	0.900	3.3±0.6	3.6±1.2	0.853	0.979
HbA1c (%)	6.5±0.3	6.9±0.4	0.396	6.8±0.1	6.8±0.3	0.816	0.118
γGTP (IU/L)	50.9±6.	47.3±6.0	0.695	72.2±8.0	63.8±6.2	0.430	0.497
AST (IU/L)	48.0±7.6	50.1±9.6	0.864	42.4±5.6	33.4±6.1	0.311	0.328
ALT (IU/L)	69.7±12.1	73.0±14.5	0.865	64.2±12.3	48.0±10.9	0.352	0.161
Free Fatty Acid (mEq/L)	0.54±0.06	0.58±0.07	0.668	0.59±0.20	0.57±0.17	0.948	0.822
Total Cholesterol (mg/dL)	185.0±15.3	186.1±20.2	0.965	146.2±22.5	157.8±25.8	0.743	0.470
Triglyceride (mg/dL)	168.0±36.5	175.0±29.1	0.876	101.0±26.0	104.0±14.1	0.927	0.922
HDL-C (mg/dL)	39.1±2.5	40.4±3.3	0.762	40.8±4.2	45.8±5.5	0.490	0.345

Data are expressed as means ± SEM

^{a}*p* value for the intergroup comparison (baseline vs 6 months)

^{b}*p* value for the intergroup comparison (changes from baseline between groups)

HDL-C, HDL-cholesterol

Table S3. Comparison of the mechanisms of action of astaxanthin and vitamin E.

	Astaxanthin	Vitamin E
**Steatosis and lipid metabolism**		
Lipid accumulation	↓↓	↓
Lipogenesis	↓↓	↓
Lipid uptake	↓	→
Fatty acid oxidation	→	↑
Lipid peroxidation	↓	↓
**Inflammation and insulin resistance**		
Macrophage/Kupffer cell accumulation	↓↓	↓
Macrophage polarization	↓↓M1, ↑↑M2	↓M1, ↑M2
T-cell accumulation	↓	↓
Glucose tolerance and insulin sensitivity	↑	→
**Fibrosis**		
Stellate cell activation	↓↓	↓
Fibrogenesis	↓↓	↓

↓, Decreased or suppressed; ↑,increased or enhanced; →, unaffected.

Table S4. Primer sequence used for real-time PCR.

Gene	5' Primer	3' Primer
Srebp1c	GGA GCC ATG GAT TGC ACA TT	GGC CCG GGA AGT CAC TGT
Lxra	CTG CAG GAC AAA AAG CTT CC	CCC TTC TCA GTC TGC TCC AC
Chrebp	GTC CGA TAT CTC CGA CAC ACT CTT	GTC CGA TAT CTC CGA CAC ACT CTT
Fasn	AGA GAC GTG TCA CTC CTG GAC TT	GCT GCG GAA ACT TCA GAA AAT
Scd1	CAT CAT TCT CAT GGT CCT GCT	CCC AGT CGT ACA CGT CAT TTT
Acc	TGG AGA GCC CCA CAC ACA	TGA CAG ACT GAT CGC AGA GAA AG
Dgat1	GTGCACAAGTGGTGCATCAG	CAGTGGGATCTGAGCCATC
Ppara	GAG GGT TGA GCT CAG TCA GG	GGT CAC CTA CGA GTG GCA TT
Cpt1a	AAA CCC ACC AGG CTA CAG TG	TCC TTG TAA TGT GCG AGC TG
Lcad	TCA CCA CAC AGA ATG GGA GA	ACG CTT GCT CTT CCC AAG TA
Cd36	AATGGCACAGACGCAGCCT	GGTTGTCTGGATTCTGGA
F4/80	CTT TGG CTA TGG GCT TCC AGT C	GCA AGG AGG ACA GAG TTT ATC GTG
Tnf	AAG CCT GTA GCC CAC GTC GTA	GGC ACC ACT AGT TGG TTG TCT TTG
Ll1b	CTG AAC TCA ACT GTG AAA TGC CA	AAA GGT TTG GAA GCA GCC CT
Il6	CCA CTT CAC AAG TCG GAG GCT TA	GCA AGT GCA TCA TCG TTG TTC ATA C
Tgfb1	TGA GTG GCT GTC TTT TGA CG	TCT CTG TGG AGC TGA AGC AA
PAI-1	TCA GCC CTT GCT TGC CTC AT	TCA GCC CTT GCT TGC CTC AT
Col1a1	ACG TCC TGG TGA AGT TGG TC	CAG GGA AGC CTC TTT CTC CT
a-SMA	TGT GCT GGA CTC TGG AGA TG	GAA GGA ATA GCC ACG CTC AG
Ccl5	TGC CCT CAC CAT CAT CCT CAC T	GGC GGT TCC TTC GAG TGA CA
Il10	GCT CTT ACT GAC TGG CAT GAG	CGC AGC TCT AGG AGC ATG TG
Cd163	GGG TCA TTC AGA GGC ACA CTG	CTG GCT GTC CTG TCA AGG CT
Cd206	CAA GGA AGG TTG GCA TTT GT	CCT TTC AGT CCT TTG CAA GC
Cd209a	CCT GGG AGA GGA AGA CTG TG	CTT GCT AGG GCA GGA AGT TG
Chi3l3	AGA AGG GAG TTT CAA ACC TGG T	GTC TTG CTC ATG TGT GTA AGT GA
Mgl1	TGA GAA AGG CTT TAA GAA CTG GG	GAC CAC CTG TAG TGA TGT GGG
18S	AGG CCC AGA GCA AGA GAG GTA	GGG GTG TTG AAG GTC TCA AAC A

Table S5. Antibodies used in FACS analysis.

Antibody	
PerCP-Cyanine5.5-conjugated NK1.1	eBioscience
PerCP-Cyanine5.5-conjugated CD3	eBioscience
PerCP-Cyanine5.5-conjugated CD19	eBioscience
PerCP-Cyanine5.5-conjugated TER-119	eBioscience
Allophycocyanin (APC)-eFluor 780-conjugated CD45	eBioscience
Phycoerythrin (PE)-conjugated CD11c	eBioscience
eFluor 450-conjugated Ly-6G (Gr-1)	eBioscience
APC-conjugated CD8	eBioscience
eFluor 450-conjugated CD3	eBioscience
PE-conjugated NK1.1	eBioscience
Fluorescein isothiocyanate (FITC)-conjugated CD45	eBioscience
PE/Cy7-conjugated F4/80	Biolegend
Alexa Fluor 647-conjugated CD206	Biolegend
APC-conjugated Ly-6C	Biolegend
PE Texas Red-conjugated CD4	Abcam
APC-Cy7-conjugated CD11b	BD Bioscienc
PE-Texas Red-conjugated CD11b	Invitrogen
